# A closing window: Canada needs to deliver on promises on health equity and principled pragmatism

**DOI:** 10.1371/journal.pgph.0007441

**Published:** 2026-10-08

**Authors:** Shashika Bandara, Muriel Mac Seing, Charity Oga-Omenka, Michelle Amri, Zahid A. Butt, Amelia Dinh, Karina Dubois-Nguyen, Marie Dorey, Joelle Ducharme, Saad El-Din Hassan, Janet Hatcher-Roberts, Justus Holben, Tina Lines, Nihad Mahmalat, Margaret Mutumba-Nakalembe, Hannah Neufeld, Nitika Pant Pai, Sanskruti Patel, Claudia Ximena Robayo-Gonzalez, Bukola Salami, Lauren Smith, Sarah Stecko, Eric Tchouaket Nguemeleu, Kathryn E. Tiedje, Bilkis Vissandjée

**Affiliations:** 1 Department of Global Population Health, Jeffrey Cheah School of Medicine and Health Sciences, Monash University, Subang Jaya, Malaysia; 2 School of Public Health, Université de Montréal, Montréal, Canada; 3 School of Public Health Sciences, University of Waterloo, Waterloo, Canada; 4 School of Population and Public Health, University of British Columbia, Vancouver, British Columbia, Canada; 5 Canadian Association for Global Health, Ottawa, Canada; 6 Department of International Development Studies, Faculty of Arts and Social Sciences, Dalhousie University, Halifax, Canada; 7 Unité de santé internationale, Université de Montréal, Montréal, Canada; 8 Canadian Red Cross, Ottawa, Canada; 9 Bruyère Health Research Institute, University of Ottawa, Ottawa, Canada; 10 Canadian Network for Neglected Tropical Diseases, Ottawa, Canada; 11 Faculty of Medicine and Health Sciences, McGill University, Montréal, Canada; 12 Schulich School of Medicine & Dentistry, Western University, London, Canada; 13 Department of Medicine, McGill University, Montréal, Canada; 14 Cumming School of Medicine, University of Calgary, Calgary, Canada; 15 Department of Community Health Sciences, University of Calgary, Calgary, Canada; 16 Dalla Lana School of Public Health, University of Toronto, Toronto, Canada; 17 Centre interdisciplinaire de développement international en santé, Université de Sherbrooke, Longueuil, Canada; 18 Department of Nursing, Université du Québec en Outaouais, Saint-Jérôme, Canada; 19 Department of Microbiology and Immunology, The Peter Doherty Institute for Infection and Immunity, The University of Melbourne, Melbourne, Australia; 20 School of Nursing, Université de Montréal, Montréal, Canada; PLOS: Public Library of Science, UNITED STATES OF AMERICA

Calls for Canada to lead in global health as a middle power are not new [1]. However, rarely have the stakes been higher with equity and human rights under attack [2]. A polycrisis is raging across the world, including a genocide in Gaza recognized by an Independent International Commission established by the UN Human Rights Council, UN recognized ‘hallmarks of genocide’ in Sudan, highest number armed conflicts since World War II, rising climate-crisis-driven disasters, continued disease outbreaks, and a rupture in global health governance worsening gender, racial, and other aspects of health equity [2–6]. Inability to prevent domestic crises such as natural disasters, weakening democracies, outbreaks weakening health systems, limit our capacity to build healthier societies, globally [2,3]. Convergence of these crises are widening existing inequities and costing lives, underscoring the urgency for Canada to reconsider its position in global health [7].

The political imperative for increased Canadian commitment to global health is clear and the policy window is open, for now. In this essay, as the steering committee of the 32^nd^ Canadian Conference on Global Health 2026 (CCGH 2026), with the theme “Collectively reclaiming global health equity: Restoring commitment to action in a fragmented and uncertain world”, we outline ways of leveraging existing capacity and intent into seizing the current window of opportunity to fulfill Canada’s commitment to global health objectives. These objectives include urgently upholding rules-based international order [8] and the ten-year global health commitments (2020–30), which includes support for sexual and reproductive health rights, strengthening Canada’s leadership in global health and gender equality, and advancing the UN Sustainable Development Goals [9].

In *The Lancet* series published in 2018, Canadian experts, along with the then Prime Minister Justin Trudeau, called for advancing a feminist international development strategy, improving the health of Indigenous communities, strengthening health systems, and strengthening principled leadership in global health [1,10]. In the opening commentary titled “Canada’s time to act”, Jocalyn Clark and Richard Horton, argued for the necessity for Canada to move forward on health more boldly, be more action-oriented, and called for greater financial investment from Canada [1]. In 2025, a joint report released by the Royal Society of Canada and the Canadian Academy of Health Sciences called on Canada to seize the opportunity to lead in a world where global health funding, multilateralism, and public health efforts such as vaccination are all under attack [7,11]. The report also called on Canada to learn from the mistakes made during the COVID-19 pandemic including hoarding vaccines, challenges in pandemic response for marginalized groups, including Indigenous communities, and lack of leadership on the global stage [11].

Perhaps the strongest recent opportunity for Canada to act on these policy recommendations arrived when Prime Minister Mark Carney declared at the World Economic Forum in January 2026 that Canada is “taking the sign down from the window” and ending the pretense that all is well [8]. In the same speech, he announced a commitment to principled pragmatism in which commitments to globally agreed upon rights, treaties, and international norms will be valued. It is crucial to follow these political declarations with evidence-informed policy processes and sustained funding commitments. Yet, in November 2025, Canada announced a reduction of funding to international aid and global health programs of CAD $ 2.7 billion over the next four years, undoing, in budget form, what had been promised [12].

As Global South countries show clear intent and commitment toward owning agenda setting in global health, in the aftermath of nationalistic retreat of Global North countries, Canada has a choice about the kind of partner it will be. First, a country that asks the world to reclaim equity cannot credibly do so while inequities endure within its own borders, including health inequities impacting Indigenous communities [7]. Acknowledging Canada’s own shortcomings can set a positive example and is a prerequisite for genuine partnership. Such honesty lends moral weight to Canada’s voice globally rather than diminishing it. Second, Canada can go against the grain and decline the trend of high-income countries retreating from global health and prioritize allyship with the Global South, while prioritizing its own marginalized communities. Genuine allyship means strengthening Canada’s financial and policy commitments to multilateralism and to its own global health community, including in research, training, and practice [13]. It is crucial that the government support locally led priorities, strengthening institutions and research capacity including increased funding for researchers and graduate students, investing in long-term and reciprocal global and local partnerships, to ensuring greater equity in how global health initiatives are designed and governed.

Therefore, we posit two main asks from the government: a) a renewed commitment to global health partnership building committing as an ally to work with Global South nations both within multilateral and bilateral mechanisms; and b) back this renewed political commitment by strengthening the funding envelope for health and determinants of health research beyond those related to defense both domestically and globally. This approach can sustainably address many rotating urgent priorities based on the latest crisis event, ensuring equity in preparedness and accountability for actors involved.

Although government commitment is necessary and funding remains crucial, the second major responsibility lies with the global health community within Canada and at large. This responsibility includes countering attacks on equity, ensuring that, as academic, civil society organizations, and other institutions, we do not retreat from our stated missions of upholding commitments toward the right to health, equity, and freedom of speech. This means standing by individuals, organizations, and communities, including students and practitioners who are advocating and pushing for human rights, against attacks on healthcare and the right to health, both within Canada and beyond its borders. This also means that, as a community, we tap into the courage required to open spaces for difficult discussions on how we can navigate this polycrisis. We cannot afford to retreat into comfortable spaces with the intention of surviving the times and advancing careers. A collective call to action by remains necessary to meaningfully chart the path of the future of global health that will define the future of our generation and generations to come. Canada needs to tackle this polycrisis head-on – not to create another hegemony, but to realize the potential of a more equitable global health system – recognizing the privileges it holds as a country and as a community within.

**Action pathways for the government and the global health community in Canada**

**Government**

- Visibly commit to a renewed stronger commitment to global health partnership building, with dedicated pathways as an ally to work with Global South nations both within multilateral and bilateral mechanisms.
- Back this renewed political commitment by strengthening the funding envelope for health, determinants of health, and health equity research, beyond those connected to defense, both domestically and globally.

**Community**

- Despite growing attacks, actively commit to upholding our stated commitments toward the right to health, equity, and freedom of speech.
- Stand by individuals, organizations, and communities, including students and practitioners who are advocating and pushing for human rights, against attacks on healthcare and the right to health, both within Canada and beyond its borders.
- Commit – tap into the courage required – to having difficult discussions, where we may disagree, on how we can navigate this polycrisis, without retreating to comfortable spaces.

As a necessary first step in building towards meaningful change, we need to bring the community together to understand the different perspectives at play; then discuss ways forward by enacting intersectional solidarity and reviving public accountability among our community of global health practitioners, researchers, trainees, policy-makers, and humanitarians alike [14]. Building upon past CCGH editions [15], at CCGH 2026, we offer spaces where we can collectively discuss pathways forward in addressing upstream determinants of global health, exploring equity in action, and sharing practical approaches and tools for implementation and accountability. While we propose a pathway to coalesce these conversations and efforts, we also recognize that a long, challenging, yet transformative journey lies ahead. Our goal is to build a better future where the health, wellbeing, and dignity of all humans are meaningfully upheld. We hence urge everyone, ourselves included, to seize this opportunity to drive change, because the time to act is now.
